# The prevalence of post-traumatic stress disorder among emergency medical services personnel in Saudi Red Crescent Authority, Riyadh, Saudi Arabia

**DOI:** 10.3389/fpsyt.2024.1391586

**Published:** 2024-05-08

**Authors:** Sattam Zaid Alanazi, Ali Abusharha, Tayyaba Afsar, Janeen H. Trembley, Suhail Razak

**Affiliations:** ^1^ Department of Optometry, College of Applied Medical Sciences, King Saud University, Riyadh, Saudi Arabia; ^2^ Department of Community Health Sciences, College of Applied Medical Sciences, King Saud University, Riyadh, Saudi Arabia; ^3^ Masonic Cancer Center, University of Minnesota, Minneapolis, MN, United States; ^4^ Minneapolis VA Health Care System Research Service, Minneapolis, MN, United States; ^5^ Department of Laboratory Medicine and Pathology, University of Minnesota, Minneapolis, MN, United States

**Keywords:** post-traumatic stress disorder (PTSD), prevalence, Saudi Arabia, gender difference, emergency medical services (EMS), occupation, working hours

## Abstract

**Background:**

Determining the prevalence of PTSD and contributing variables among (EMS) specialists was the goal of the current investigation. Furthermore, limited evidence exists regarding the application of PCL-5 for EMT practitioners, and the incidence of PTSD among different age groups and genders in Saudi Arabia.

**Methods:**

This cross-sectional descriptive study includes 211 prehospital care providers of the Saudi Red Crescent Authority stations in Riyadh. The randomization was done using Google Forms into subgroups according to participants’ gender, years of experience, occupations, and average working hours. The presence and severity of PTSD symptoms were evaluated using the 20-item PTSD Checklist for DSM-5 (PCL-5) self-report questionnaire. Data were analyzed using Pearson Chi-Square, Mann-Whitney and Kruskal-Wallis tests. The reliability statistics were calculated using Cronbach’s Alpha for the 20-survey questionnaire.

**Results:**

The comparison of PCL-5 total scores indicated more PTSD symptomatology among females (1.61 + 0.799) as compared to male workers (1.13 + 0.642). The total score of PTSD demonstrated no statistically significant (P=0.79) differences between our age group classifications. In terms of the participants’ city (Riyadh), the total PTSD score was less than the cutoff point which is 31. PTSD total score may not be affected by working experience as indicated by the non-significant difference in prevalence among EMT practitioners having <5 years, 5-10 years and above 10 years of working experience (P=0.215 with X2 = 3.076). PTSD incidence is affected by the type of occupation as statistically significant differences between groups (P=0.001) were recorded depending on the position and responsibilities of EMS practitioners. PTSD is also affected by average working hours per week, and there were statistically significant differences between groups (P=0.001).

**Conclusion:**

The total score of PTSD in the case of emergency service practitioners was found to be 33.7% among all the research participants, which may be regarded as a high prevalence when compared to the general population. Our investigations would contribute to a better understanding of the underlying factors of mental stress in EMS specialists in Saudi Arabia and to the development of adequate mental health practices.

## Introduction

Post-traumatic stress disorder (PTSD) is characterized by directly experiencing traumatic events or repeatedly witnessing, learning, or experiencing the details of these events (1). Many factors increase the risk of PTSD, including the severity of the trauma and threat to life during the event, as well as post-traumatic factors such as inadequate coping mechanisms, lack of social support, and frequent exposure to trauma (1).

Emergency medical technicians (EMTs) and Emergency medical services (EMS) personnel experience stress from dealing with trauma, serious illnesses, disabilities, or fatalities. (2). In addition, regular exposure to potentially stressful situations for an extended period may raise the risk of severe psychiatric impairments. PTSD may be a significant issue that has an impact on both the health of EMS and EMT workers and the quality of patient treatment (3). According to the Jonsson et al. (4) study, nearly 62% of paramedic practitioners in emergency teams experienced a traumatic event during their work, most often linked with casualties involving children (4). A study done by Al Enazi and AlEnzie (5) in Saudi Arabia has shown that stress and tension among paramedics are the main factors concerning poor performance, and the leading cause of the incapability to perform assigned tasks. In contrast, not many studies have been conducted to measure the prevalence of PTSD among paramedics in Saudi Arabia. However, the prevalence of PTSD worldwide was 10% in 2012 (6). In the USA, the prevalence is about 8.7%, while in European, Asian, and African countries it is lower (1, 6). According to Alaqeel et al. (7), 26% of EMS personnel in Saudi Arabia screened positive for PTSD. Attention should be directed to this issue through regular psychological assessment and the implementation of psychological rehabilitation programs for EMS personnel. The study relates to only one city center, so generalization would not be appropriate.

At the professional level, poor psychological health can lead to vulnerability to making clinical mistakes, communication failure, diminished clinical capability (8), absenteeism, poor job performance (9), and increased turnover (10). Such undesirable consequences resulting from impaired psychological health can impact the quality of patient care and safety. Consequently, healthcare professionals must identify mental illness early, to address these issues (7). Experiencing a traumatic event is not the only factor causing PTSD among individuals. Thus, identifying risk factors other than exposure to traumatic events, such as personal and occupational characteristics that may predict the development of PTSD, may lead to further effective management and control of prehospital emergency stress in emergency responders (11).

Considering that EMTs generally have the highest prevalence of PTSD across prehospital healthcare practitioners (11-35%) (11, 12), it is imperative to evaluate the mental health of EMTs and identify staff members who are at a high risk of getting PTSD (11, 13). Emergency medical services personnel in Saudi Arabia are not aware of the significance and the occurrence of PTSD among them, its effect on their mental well-being, and the quality of patient care. EMS personnel need to be aware of stress management and PTSD symptoms to provide quality care. Lack of knowledge can lead to burnout, decreased productivity, and early retirement. Proper training can help them cope with job challenges and maintain their well-being. Consequently, this situation may harm their mental well-being which may diminish enthusiasm and lower the quality of patient care. Therefore, the purpose of this study is to measure the prevalence of PTSD among emergency medical services personnel in Riyadh, Saudi Arabia, and to determine the main factors contributing to PTSD. We assumed that paramedics are frequently subjected to traumatic situations and could have higher PTSD prevalence rates than the general population. The scarcity of published studies highlights a critical need for further research to better understand the magnitude of PTSD among EMS practitioners. It is crucial to have a good understanding of the prevalence and incidence of PTSD, as well as effective methods for managing it. Conducting studies in the Middle East and Eastern world is of paramount importance to obtain a comprehensive understanding of mental health awareness and the means of overcoming psychological challenges. To our knowledge, no studies have been conducted to measure the prevalence of PTSD among paramedics in Saudi Arabia. This research will help assess the extent of psychological stress among emergency medical services personnel encountered in the prehospital environment. The assessment of PTSD total score may benefit stakeholders in addressing ways to decrease the level of stress among emergency medical services practitioners to improve the quality of patient care in addition to protecting the mental health of healthcare providers.

## Material and methods

### Ethical approval

Ethical approval was obtained from the Institute Review Board of King Saud University (IRB). A consent form was attached to the questionnaire stating the purpose of the study and the contact information of the main author for any questions and/or inquiries. The identity of the participant was not obtained.

### Design and setting

**Figure 1:**
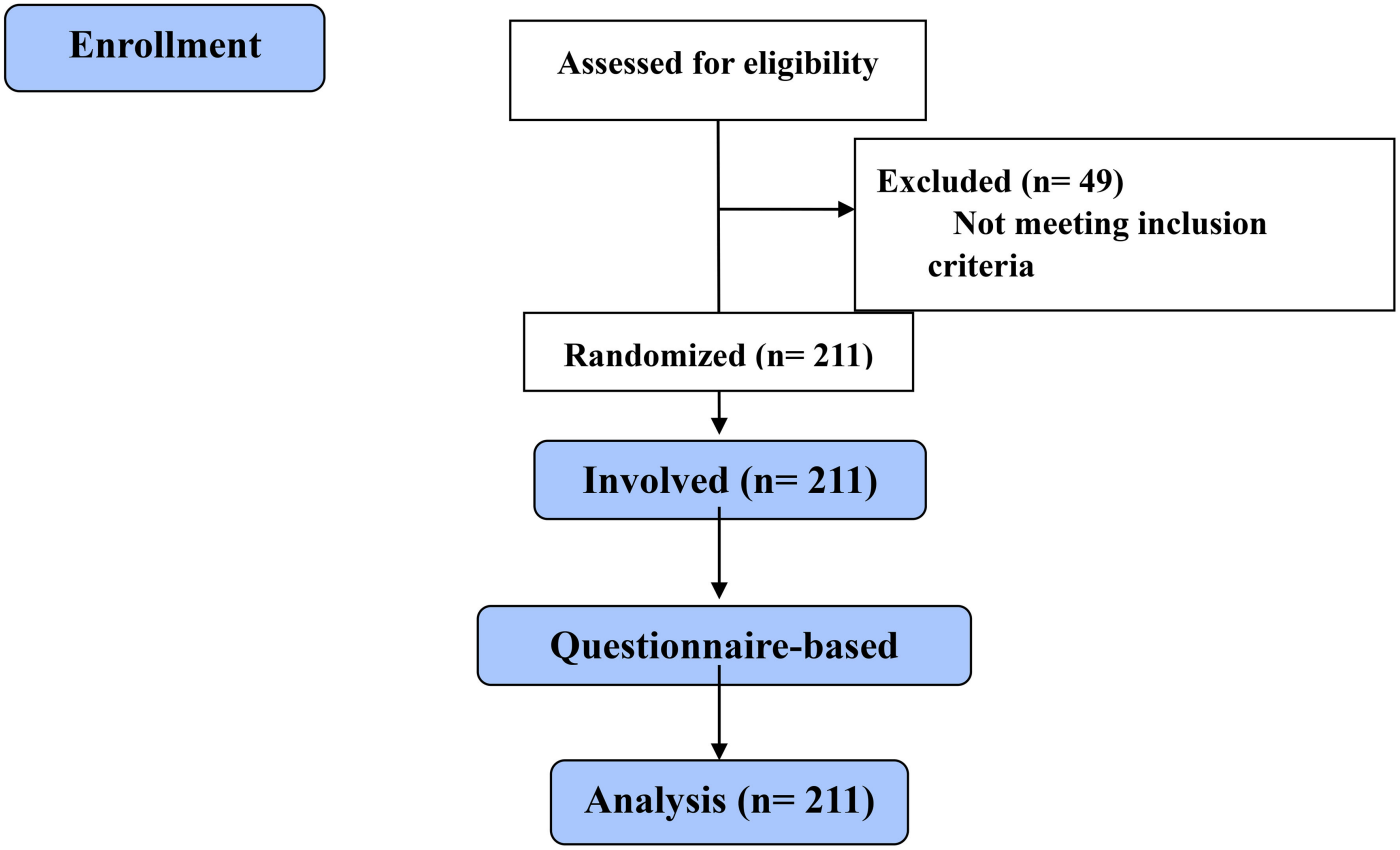
Consort diagram of participant’s flow.

This study is a cross-sectional descriptive study. A descriptive study using a questionnaire-based survey (PCL-5 on emer). Prehospital care providers (Physicians, Paramedics, Emergency Medical Technicians, and Emergency Medical specialties) of the Saudi Red Crescent Authority stations in Riyadh. **Figure 1** summarizes the study design.

### Recruitment

This is a descriptive study using a questionnaire-based survey. The questionnaire was adopted from the PTSD Diagnostic Scale for DSM-5. The total population in EMS departments and stations are 350 male and female practitioners. The sample size was calculated using the EPI program based on the following: a confidence interval of 95% and a 5% margin of error. The sample size obtained was 184. The calculated sample size of 184 was based on a power analysis conducted during the study design phase. Based on these parameters, the sample size calculation indicated that a minimum of 184 participants would be required to achieve adequate statistical power for our study objectives. However, in our study, 211 practitioners participated. The questionnaires were administered to all prehospital care providers (Physicians, Paramedics, Emergency Medical Technicians, Emergency Medical specialists, and intern students in Riyadh, Saudi Arabia. The PTSD total score represents the sum of individual scores on a standardized assessment tool, such as the PTSD Checklist for DSM-5 (PCL-5) or the Clinician-Administered PTSD Scale (CAPS). Each participant’s responses to the items on the assessment tool are assigned numerical values, and the total score is obtained by summing these values. A higher total score indicates a greater overall severity of PTSD symptoms. On the other hand, the PTSD mean score is calculated by dividing the total score by the number of items on the assessment tool. This provides an average score that represents the average severity of PTSD symptoms experienced by the participants. It can be useful for comparing the average symptom severity among different groups or populations. Both the PTSD total score and mean score are quantitative measures that provide insights into the severity of PTSD symptoms experienced by the participants in our study. These scores help us quantify and understand the level of PTSD symptomatology within the sample.

### Randomization and approaching

Participants were randomly allocated to different groups without consideration of any specific characteristics or variables. This was done to ensure an unbiased distribution of participants across the groups, reducing the potential for confounding factors. The randomization process was conducted using a randomization feature available in Google Forms. When participants accessed the online survey link, they were directed to the Google Forms platform, where they were presented with the survey questions. No stratification was employed in our randomization process. The randomization was done automatically using Google Forms into subgroups according to participants’ cities, genders, years of experience, occupations, and average working hours. An Interview-based questionnaire was conducted by three interviewers, training was done in multiple sessions and was followed by the piloting stage and final evaluation and recommendation.

### Inclusion/exclusion criteria

We targeted all healthcare facilities in Riyadh that provide emergency medical services with no exceptions. Riyadh is the capital city with a population of almost 8 million people that is served by only 110 Emergency centers. These services include emergency medical services, trauma care, critical care, or other specialties that are typically associated with the management of emergencies. These centers are characterized by high patient volume. To provide a clearer understanding of the study population, we targeted all healthcare facilities in Riyadh that meet the criteria of providing emergency medical services or trauma care. By including all such facilities, we aimed to capture a comprehensive representation of healthcare providers working in these settings and their experiences with traumatic events. Emergency Medical Services providers working as administrative EMS were excluded from the study. The decision to exclude administrative emergency center workers was based on several factors related to the specific objectives and scope of our study. Here are the justifications for this exclusion:

1. Focus on Direct Patient Care Providers: Our study aimed to assess the incidence of post-traumatic stress disorder (PTSD) symptoms among healthcare providers directly involved in emergency medical services and trauma care. By focusing on direct patient care providers, we aimed to capture individuals who have firsthand exposure to traumatic events and are more likely to experience work-related stressors associated with emergency care provision.
2. Different Nature of Roles: Administrative emergency center workers typically have different job responsibilities and may not be exposed to the same level of direct patient care and trauma-related experiences as frontline healthcare providers. Their roles often involve administrative duties, management tasks, or support functions that are distinct from the direct provision of emergency medical services. Including administrative workers may introduce heterogeneity in the sample and potentially dilute the specific focus on direct patient care providers.

### PTSD checklist for DSM-5 (PCL-5)

The data collection process involved the identification, approaching, and recruitment of subjects. The identification process is related to all pre-hospital care providers. The presence and severity of PTSD symptoms are evaluated using the 20-item PTSD Checklist for DSM-5 (PCL-5) self-report questionnaire (14). The PTSD Diagnostic Scale for DSM-5 is a widely recognized and validated instrument for assessing post-traumatic stress disorder symptoms according to the criteria outlined in the Diagnostic and Statistical Manual of Mental Disorders, Fifth Edition (DSM-5). As our study aimed to assess the prevalence of PTSD symptoms among EMS personnel in Saudi Arabia, we chose this questionnaire due to its well-established psychometric properties and applicability to our research objectives. It evaluates four DSM-5 symptom clusters, B to E, including intrusion (five items; B), avoidance (two items; C), negative cognitive and mood alterations (seven items; D), and changes in arousal and reactivity (alterations in arousal and reactivity) (six items; E) (14). The items are rated from 0 (not at all) to 4 (extremely) and are summed for a total severity score (15–18). The PCL-5 can determine a provisional diagnosis by Summing all 20 items (range 0-80) and using a cut-point score of 31-33 appears to be rational for the presence of PTSD. The lower the cutoff score, the more lenient the criteria for inclusion, increasing the possible number of false-positives. The higher the cutoff score, the more stringent the inclusion criteria and the more potential for false-negatives (19). In this study, scores on PCL5 showed strong internal consistency (α = 0.94), test-retest reliability (r = 0.82), and convergent (rs = 0.74 - 0.85) and discriminant (rs = 0.31 - 0.60) validity. Several studies have supported these psychometric properties in different populations (20–22). Data was saved in the investigator’s laptop with password protection, meaning that only the researchers had access to the information.

### Statistical analysis

Data are presented as mean ± SD. Data were analyzed using SPSS version 20.0. Descriptive statistics Mean and standard deviation were calculated for all variables. The normal distribution for the data was tested using the Shapiro-Wilks test, Observation of histograms, and Q-Q plots performed to determine whether the variables were normally distributed. Data were analyzed using Pearson Chi-Square, Mann-Whitney and Kruskal-Wallis tests. test was used to compare the prevalence of all categorical variables including city, age, gender, Workplace, Occupation, and Average hours worked per week. **Table 1** represents the number of participants screened positive or negative for PTSD symptoms. The P values less than 0.05 were considered statistically significant with an estimated 95% confidence interval (CI).

**Table 1:** Represents the number of participants screened positive for PTSD symptoms.

Characteristics				PTSD-NO.%		X^2^ -value	P value
		N	%	Present	Absent		
**City**	**Riyadh**	205	97.2	53 (25.9)	152 (74.1)		
**Gender**	**Male**	191	90.5	47 (24.6)	144 (75.4)	3.862	0.049
	**Female**	20	9.5	9 (45)	11 (55)		
**Age**	**20-30**	66	31.3	17 (25.8)	49 (74.2)	0.434	0.805
	**30-40**	120	56.9	31 (25.8)	89 (74.2)		
	**> 40**	25	11.8	8 (32)	17 (68)		
**Nationality**	**Saudi**	211	100	56 (26.5)	155(73.5)	-	-
**Workplace**	**SRCA**	206	97.6	53 (25.7)	153 (74.3)	2.941	0.086
	**Other**	5	2.4	3 (60)	2 (40)		
**Occupation**	**Physician**	3	1.4	2 (66.7)	1 (33.3)	11.768	0.008
	**EMS**	75	35.5	25 (33.3)	50 (66.7)		
	**EMT**	129	61.1	26 (20.2)	103 (79.8)		
	**Intern student**	4	1.9	3 (75)	1 (25)		
**Working hours**	**30-40 hours**	37	17.5	12 (32.4)	25 (67.6)	15.190	0.001
	**41-50 hours**	144	68.2	29 (20.1)	115 (79.9)		
	**51-60 hours**	27	12.8	15 (55.6)	12 (44.4)		
	**61-70 hours**	3	1.4	0	3 (100)		
**experience**	**< 5 years**	40	19	15 (37.5)	25 (62.5)	3.076	0.215
	**5-10 years**	94	44.5	22 (23.4)	72 (76.6)		
	**>10**	77	36.5	19 (24.7)	58 (75.3)		

## Results

### Reliability statistics

The reliability statistics were calculated using Cronbach’s Alpha for the 20-survey questionnaire as being 0.910 for a sample of this size and containing 20 items which are considered highly reliable (**Table 2**).

**Table 2:** Demographic characteristics of the study population.

Cronbach’s Alpha	N of Items	Number of cases	%
**0.910**	20	211	100

**Figure 2:**
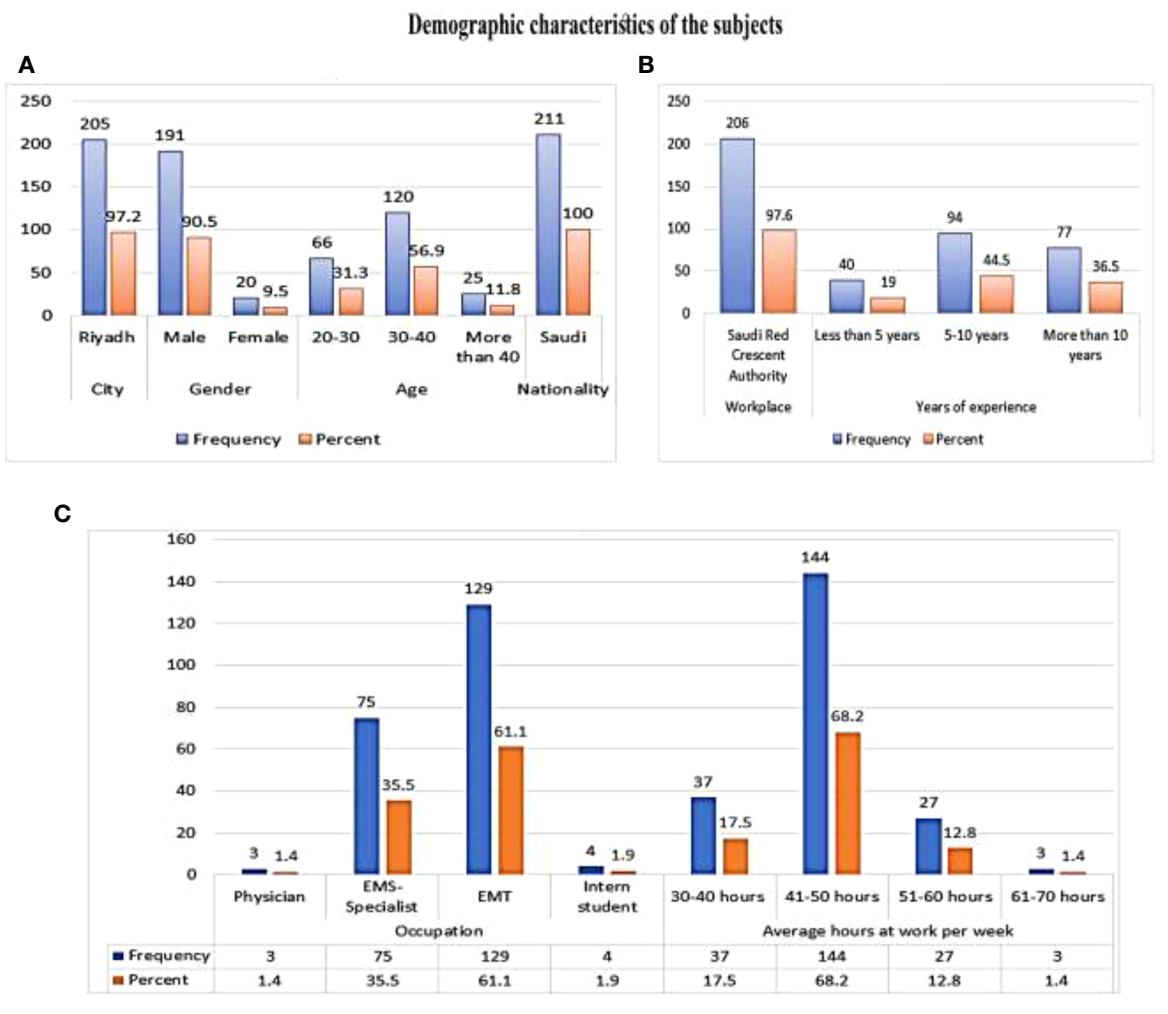
Demographic characteristics of the study participants.

**Figure 2** presents the demographic characteristics of the study population. In this study, emergency medical services personnel in the Saudi Red Crescent Authority involving 205 individuals representing 97.6% of the total sample, together with 5 from other Authorities (2.4%), participated in our research. Males constituted the major part of the study population with a total of 191 individuals (90.5%) while 20 females also participated (9.5%). In terms of experience in the field, 40 participants (19%) had less than 5 years, 94 (44.5%) had between 5 and 10 years, and 77 (36.5%) had more than 10 years.

### PTSD score/demographic and professional characteristics

The total PTSDS score and the mean PTSD score, together with demographic and professional characteristics, are shown in **Table 3**. The male total score of PTSDS was 22.67 + 12.85, while 32.35 + 15.88 for females with an average score of PTSDS 1.13 + 0.642 and 1.61 + 0.799 respectively. The comparison between the male and female was statistically significant P=0.007. Regarding the participant’s age, the total score was 24.18 ± 13.6 for the age 20-30 years while 23.01 + 13.49 for 30-40 years, and for more than 40 years the total PTSD was 24.8 + 14.23. While the average PTSD score was 1.20 + 0.65 for the ages 20-30 while 1.15 ± 0.67 for 30-40 age and more than 40 years the total PTSD score was 1.24 + 0.711. In terms of the participants’ city (Riyadh), the total PTSD score was 23.38 + 12.96 with an average of 1.16 + 0.64. The score is less than the cutoff point which is 31. Consequently, we can assume that the incidence is small in the population. Regarding the years of experience, the total PTSD score for less than 5 years was 28.15 + 15.99, for 5-10 years of experience was 21.6 + 11.63, and for more than 10 years the score was 23.55 + 13.6, the PTSD mean was 1.4, 1.8, and 1.17 respectively. In the occupation group, the average was 1.85 + 0.78 for physicians representing 46.25%, 1.3 + 0.7 for EMS specialists 33.13%, 1.04 + 0.58 for the EMT group 26.16%, and 2.33 + 1.03 for intern students representing 55.94%. The total PTSD was (37 + 15.7, 26.5 + 14.1, 20.93 + 11.6, and 44.75 + 20.71) respectively. The total score for PTSD among employees who work for 30-40 hours per week (36.14%) was 28.91 + 15.77, with an average of 1.44 + 0.78. For those working 47-50 hours per week (25.81%) it was 20.65 + 10.76, with an average of 1.03 + 0.53. Regarding those working 51-60 hours per week (41.25%) the results showed that the total PTSD was 33 + 16.7, with an average of 1.65 + 0.83. The final classification in the range of 61-70 hours per week (17.91%) the score was 14.33 + 9.29, with an average of 0.71 + 0.46.

**Table 3:** Relationship between demographic and professional characteristics with PTSD score and PTSD score mean.

		N	%	B	C	D	E	PTSD Total	SD	%	PTSD (Average)	SD	P	TEST
**City**	**Riyadh**	205	97.2	5.06	2.29	8.24	7.77	23.38	12.96	29.23	1.16	0.64	-	-
**Gender**	**Male**	191	90.5	5.03	2.24	7.95	7.43	22.67	12.85	28.34	1.13	0.642	0.007	MANN WHITNEY
	**Female**	20	9.5	7.05	3.40	11.10	10.80	32.35	15.88	40.44	1.61	0.794		
**Age**	**20-30**	66	31.3	5.45	2.53	8.33	7.86	24.18	13.16	30.23	1.20	0.65	0.799	Kruskal-Wallis Test
	**30-40**	120	56.9	5.08	2.26	8.10	7.56	23.01	13.49	28.76	1.15	0.67		
	**> 40**	25	11.8	5.32	2.32	8.80	8.36	24.8	14.23	31.00	1.24	0.711		
**Nationality**	**Saudi**	211	100	5.22	2.35	8.25	7.75	23.59	13.43	29.49	1.17	0.67	-	-
**Workplace**	**SRCA**	206	97.6	5.08	2.32	8.17	7.75	23.33	13.3	29.16	1.16	0.66	0.081	MANN WHITNEY
	**Other**	5	2.4	11.20	3.60	11.40	7.80	34	14.26	42.50	1.67	0.66		
**Occupation**	**Physician**	3	1.4	5.66	2.00	14.66	14.66	37	15.7	46.25	1.85	0.78	0.001	Kruskal-Wallis Test
	**EMS**	75	35.5	6.16	2.7467	9.16	8.44	26.5	14.1	33.13	1.3	0.7		
	**EMT**	129	61.1	4.48	2.05	7.32	7.06	20.93	11.6	26.16	1.04	0.58		
	**Intern** **student**	4	1.9	11.25	5.00	16.50	12.00	44.75	20.71	55.94	2.23	1.03		
**Working hours per week**	**30-40 hours**	37	17.5	5.66	2.00	14.66	14.66	28.91	15.77	36.14	1.44	0.78	0.001	Kruskal-Wallis Test
	**41-50 hours**	144	68.2	6.16	2.74	9.16	8.44	20.65	10.76	25.81	1.03	0.53		
	**51-60 hours**	27	12.8	4.48	2.05	7.32	7.06	33	16.7	41.25	1.65	0.83		
	**61-70 hours**	3	1.4	11.25	5.00	16.50	12.00	14.33	9.29	17.91	0.71	0.46		
**experience**	**< 5 years**	40	19	6.67	3.05	9.57	8.85	28.15	15.99	35.19	1.40	0.799	0.083	Kruskal-Wallis Test
	**5-10 years**	94	44.5	4.63	2.05	7.61	7.37	21.6	11.63	27.00	1.08	0.58		
	**>10**	77	36.5	5.19	2.36	8.35	7.64	23.55	13.6	29.44	1.17	0.68		
**Average mean**		211	100	6.56	2.80	9.79	8.77	26.46	-	33.07	1.31	-	-	-
**Average SD**		-	-	2.77	1.20	3.28	2.68	8.04	-	10.06	0.40	-	-	-

### The PSTD knowledge and aptitude

**Table 4** represents the descriptive statistics for each question included in the questionnaire survey and shows the frequencies and percentages for 20 questions and the participants’ agreements in terms of Not at all (0), A little bit (1), Moderately (2), Quite a bit (3), and (4) Extremely. The mean and standard deviation were calculated for each question to measure the participants’ agreement level.

**Table 4:** The frequency of responses and the mean for each query.

Questions		Not at all	A little bit	Moderately	Quite a bit	Extremely	Mean	Std. Deviation
**1. Repeated, disturbing, and unwanted memories of the stressful experience?**	N	76	100	21	8	6	.9005	.92816
	%	36	47.4	10	3.8	2.8		
**2. Repeated, disturbing dreams of the stressful experience?**	N	70	100	25	11	5	.9621	.93528
	%	33.2	47.4	11.8	5.2	2.4		
**3. Suddenly feeling or acting as if the stressful experience were actually happening again?**	N	67	79	44	19	2	1.0995	.98297
	%	31.8	37.4	20.9	9	0.9		
**4. Feeling very upset when something reminded you of the stressful experience?**	N	58	85	41	20	7	1.2085	1.05294
	%	27.5	40.3	17.4	9.5	3.3		
**5. Having strong physical reactions when something reminded you of the stressful experience?**	N	75	81	31	16	8	1.0569	1.07198
	%	35.5	38.4	14.7	7.6	3.8		
**6. Avoiding memories, thoughts, or feelings related to the stressful experience?**	N	56	85	37	24	9	1.2654	1.10224
	%	26.5	40.3	17.5	11.4	4.3		
**7. Avoiding external reminders of the stressful experience?**	N	69	81	39	17	5	1.0900	1.02188
	%	32.7	38.4	18.5	8.1	2.4		
**8. Trouble remembering important parts of the stressful experience?**	N	59	81	42	25	5	1.2322	1.05474
	%	27.5	38.4	19.9	11.8	2.4		
**9. Having strong negative beliefs about yourself, other people, or the world?**	N	82	67	33	23	6	1.0711	1.11256
	%	38.9	31.8	15.6	10.9	2.8		
**10. Blaming yourself or someone else for the stressful experience or what happened after it?**	N	61	88	36	13	13	1.1896	1.10930
	%	28.9	41.7	17.1	6.2	6.2		
**11. Having strong negative feelings such as fear, horror, anger, guilt, or shame?**	N	78	63	41	22	7	1.1327	1.12606
	%	37	29.9	19.4	10.4	3.3		
**12. Loss of interest in activities that you used to enjoy?**	N	60	82	41	21	7	1.2085	1.06642
	%	28.4	38.99	199.4	10	3.3		
**13. Feeling distant or cut off from other people?**	N	72	70	36	25	8	1.1801	1.14050
	%	34.1	33.2	17.1	11.8	3.8		
**14. Trouble experiencing positive feelings?**	N	51	94	6	24	6	1.2417	1.03435
	%	24.2	44.5	17.1	11.4	2.8		
**15. Irritable behavior, angry outbursts, or acting aggressively?**	N	77	53	42	30	9	1.2464	1.20946
	%	36.5	25.1	19.9	14.2	4.3		
**16. Taking too many risks or doing things that could cause you harm?**	N	67	78	32	24	10	1.2038	1.14692
	%	31.8	37	15.2	11.4	4.7		
**17. Being “super alert” or watchful or on guard?**	N	64	72	41	31	3	1.2275	1.08030
	%	30.3	34.1	19.4	14.7	1.4		
**18. Feeling jumpy or easily startled?**	N	63	73	42	25	8	1.2512	1.12056
	%	29.9	34.6	19.9	11.8	3.8		
**19. Having difficulty concentrating?**	N	65	74	32	34	6	1.2512	1.14161
	%	30.8	35.1	15.2	16.1	2.8		
**20. Trouble falling or staying asleep?**	N	53	63	35	41	19	1.5735	1.29764
	%	25.1	29.9	16.6	19.4	9		

### Percent score of PTSD symptoms

**Table 5** represents the number of participants screened positive for PTSD symptoms in different demographic and professional categories. The result shows 47 (25.9%) males with a present sign and 152 (74.1%) with an absent sign, while there were 9 (45%) females with a present sign and 11 (55%) with an absent sign. The comparison was statistically significant (P=0.049 with X^2 =^ 3.862). Regarding the influence of occupation on absence and presence of signs the results show that for 2 physicians (66.7%) they were present, while absent in 1 case (33.3%). For 25 (33.3%) EMS personnel they were present, while for 50 (66.7%) they were absent. Regarding 26 (20.2%) EMT personnel, signs were present, while in the case of 103 (79.8%) they were absent. In the case of the intern students, 3 (75%) exhibited present signs, and 1 (25%) absent. The comparison was statistically significant (P=0.008 with X2 = 11.768).

**Table 5:** The percentage of participants screened positive or negative for PTSD symptoms.

Characteristics				PTSD-NO.%		X^2^ -value	P value
		N	%	Present	Absent		
**City**	**Riyadh**	205	97.2	53 (25.9)	152 (74.1)		
**Gender**	**Male**	191	90.5	47 (24.6)	144 (75.4)	3.862	0.049
	**Female**	20	9.5	9 (45)	11 (55)		
**Age**	**20-30**	66	31.3	17 (25.8)	49 (74.2)	0.434	0.805
	**30-40**	120	56.9	31 (25.8)	89 (74.2)		
	**> 40**	25	11.8	8 (32)	17 (68)		
**Nationality**	**Saudi**	211	100	56 (26.5)	155(73.5)	-	-
**Workplace**	**SRCA**	206	97.6	53 (25.7)	153 (74.3)	2.941	0.086
	**Other**	5	2.4	3 (60)	2 (40)		
**Occupation**	**Physician**	3	1.4	2 (66.7)	1 (33.3)	11.768	0.008
	**EMS**	75	35.5	25 (33.3)	50 (66.7)		
	**EMT**	129	61.1	26 (20.2)	103 (79.8)		
	**Intern student**	4	1.9	3 (75)	1 (25)		
**Working hours**	**30-40 hours**	37	17.5	12 (32.4)	25 (67.6)	15.190	0.001
	**41-50 hours**	144	68.2	29 (20.1)	115 (79.9)		
	**51-60 hours**	27	12.8	15 (55.6)	12 (44.4)		
	**61-70 hours**	3	1.4	0	3 (100)		
**experience**	**< 5 years**	40	19	15 (37.5)	25 (62.5)	3.076	0.215
	**5-10 years**	94	44.5	22 (23.4)	72 (76.6)		
	**>10**	77	36.5	19 (24.7)	58 (75.3)		

The results show that 12 (32.4%) of those working 30-40 hours per week exhibited present signs, with 25 (67.62%) exhibiting an absent sign. In the case of those working 41-50 hours per week, 29 (20.1%) exhibited present signs, and 115 (79.9%) exhibited absent signs. A total of 15 (55.6%) of those working 51-60 hours per week exhibited present signs, and 12 (44.4%) exhibited absent signs. Finally, for those working 61-70 hours per week, 3 (100%) exhibited absent signs concerning PTSD. The comparison was statistically significant (P=0.001 with X2 = 15.190).

In terms of differences in experience and incidence of PTSD, we noticed that 15 individuals (37.5%) with less than 5 years of experience exhibited a present sign and 25 (62.5%) exhibited an absent sign, while 22 (23.4%) with 5-10 years’ experience exhibited a present sign and 72 (76.6%) and absent sign. Finally, in terms of those with more than 10 years of experience, 19 (24.7%) exhibited a present sign and 58 (75.3%) an absent sign. The comparison was not statistically significant (P=0.215 with X2 = 3.076).

## Discussion

There is limited data on PTSD evaluation due to a lack of qualitative studies that allow EMS professionals to express their feelings regarding work-related stressors (4). PTSD may harm their mental well-being which in turn might diminish enthusiasm and lower the quality of patient care. There is gender specific differences in the prevalence and incidence of comorbid disorders such as depression, anxiety and PTSD. Investigations indicated that females are two-fold higher risk of developing PTSD compared to men (23). Likewise, our data indicated that female EMS practitioners tend to have a higher PTSD total score compared with male practitioners. Our study’s findings are consistent with those of Jonsson et al. (4) who undertook a meta-analysis study that shows that females may be more likely to have a higher PTSD prevalence than males, as they may have experienced more anxiety and depression (24). Similarly, Darensburg and colleagues (25) who studied 41 females and 58 males working as police officers, found that females may have a high prevalence regarding PTSD due to shouldering more responsibility in terms of their families and children. Additionally, many other factors such as physiological variables including hormone variations, the influence of reproductive events such as delivery and menopause, and more extreme emotional reactions, may make women more prone to developing PTSD (26).

Limited data is available to assess the impact of age on the tendency of PTSD occurrence (27). In current study it was recorded that PTSD total score may not be affected by age as no statistically significant differences recorded between different age group classifications (P=0.79). Regarding the Pearson chi-square test, the result shows that 25.8% aged between 20 and 30 years exhibited a present sign, 74.2% showed no signs, while 25.8% for the 30-40 group exhibited a present and 74.2% in that category have absent signs. Finally, for those aged 40 and more, 32% exhibited a present and 68% an absent sign. However, the comparison was not statistically significant (P=0.805 with X^2 =^ 0.434). Our study’s findings are consistent with those of some previous studies (28) which illustrate that PTSD prevalence may not be affected by age, the reason is unknown or unclear as yet, and needs more investigation. Norris and colleagues observed the influence of age on prevalence of PTSD in a cultural perspective, and compared the effects of age after similar catastrophes in three different regions of the world. According to their findings, age does not seem to have a noteworthy impact on PTSD prevalence. It was concluded that PTSD is more depended upon social, economic, cultural, and historical context of the disaster-affected region than age (29). Contrary to Our findings, Darensburg et al. (25) reported that PTSD symptoms tends to increase with age due to having more responsibilities toward work, job position, and family. In addition, Glaesmer and coworkers presented that any difference may be due to impaired physical health and quality of life as a result of ageing (30). We believe that the stress and trauma that younger health professionals encounter on the job may put them at a higher risk of acquiring PTSD. This could be a result of their inexperience, lack of coping skills, and initial exposure to stressful situations. However, due to repeated exposure to stressful events throughout their employment, older, longer-tenured health professionals may also be at risk of acquiring PTSD. Health professionals of all ages should be aware of the symptoms and indications of PTSD and seek assistance if necessary.

In terms of city, the score was less than the cutoff point so we can assume that the prevalence is small in the population. However, we cannot make a generalized statement as some neighborhoods may experience fewer urgent situations that need emergency services practitioners’ involvement as a result of the good quality of life. In addition, the large number of practitioners in each hospital in cities such as Riyadh might be an additional factor. Additionally, it may as a result of the large number of hospitals within the city. However, we recommend that more research should be undertaken to establish the situation regarding differences between cities.

Negative working conditions, such as long working hours, layoffs, workplace stress etc. can contribute to occupational PTSD (31). Our result also shows that PTSD symptomology and total score may be affected by occupational status. There were statistically significant differences between groups (P=0.001). We ascertain that healthcare practitioners may develop positive signs of PTSD with different prevalence, depending on their position and responsibilities concerning patients, serious situations such as death, or critical accidents which make them have PTSD. In addition, EMTs, EMS, and physicians may be more prone to PTSD as a result of long shifts or having to deal with critical situations daily. The average working hours per week on the part of practitioners were considered. Our results show that the PTSD total score may be affected by average working hours per week and that there were statistically significant differences between groups (P=0.001). Our findings are consistent with those of a previous study by Jackson et al. (32) who found that working hours per week may increase the prevalence of PTSD among surgical residents compared with the general population. In contrast, Li et al. (33) reported no statistically significant differences between practitioners’ working hours classification and the prevalence of PTSD during Covid-19. This variation in results is may be due to variations between hospitals and practitioners or methodological differences between the studies. However, our results support that PTSD prevalence may be affected by average working hours per week, in that there were statistically significant differences found between groups (P=0.001). Our findings might indicate a causal relationship between extended workweeks and an elevated risk of PTSD among healthcare professionals. Health professionals who work more than 40 hours per week are most likely to experience PTSD symptoms.

The capacity of healthcare professionals with greater expertise in dealing with stress and trauma over time may make them less likely to acquire PTSD. Nonetheless, regardless of their level of expertise, encountering traumatic events, including patient deaths, medical mistakes, and exposure to infectious pathogens, can still have a substantial influence on their mental health (34). Our results show that PTSD prevalence may not be affected by working experience. Certainly, no statistically significant differences were found between groups (P=0.083). The results of the current study are not consistent with those of Robinson and co-investigators, who studied police officers. They reported that years of experience in the field may increase the prevalence of PTSD compared with those of less than 11 years’ experience, due to fewer skills and less experience (35). This variation in results between this study and ours might be due to methodological differences as in our study we classified the subjects as having less than 10 years of experience. From our data, we perceived that healthcare professionals, regardless of their level of expertise, endure the possibility of suffering from PTSD. Health professionals need to prioritize their mental health and get help if they are showing signs of PTSD. Our results support the view that PTSD prevalence may not be affected by working experience.

In general, it was clear that EMTs generally have the highest prevalence of PTSD among pre-hospital healthcare practitioners (11-35%) (11, 12). Consequently, our results support this point in that the prevalence of emergency service practitioners was 33.7% among all the participants who were involved in this study. According to Alakeel and colleagues (7) who studied the prevalence among EMS in King Abdul Aziz Medical City (KAMC), found that prevalence was 26% of EMS personnel had a positive screening for PTSD. He recommended paying more attention directed toward this issue through regular psychological evaluation and implementing psychological rehabilitation programs for EMS personnel. Additionally, (36) found that the prevalence was 25% among Iranian Red Crescent volunteers. Furthermore, (37) who studied the prevalence among EMS the results illustrated that 21% showed positive signs of PTSD among ambulance service workers. Moreover, among firefighters (38) represent that the prevalence was 29.3%. Also, (35) reported that the prevalence among police officers was 13%, (39) illustrated that the prevalence was 57% among two hundred Saudi firefighters.

However, compared to the prevalence rate among other first responders (e.g., firefighters, police officers, and EMS personnel), the total score in this study seems to be high. The participants worked in healthcare units with a lot of traumatic scenes, and as was previously mentioned, they frequently encountered hardship, decomposed and dismembered bodies, terrifying and horrible scenes, and direct threats of death, among other traumatic experiences, which could account for this prevalence rate (36).

## Conclusion

This study’s objectives were to ascertain the prevalence of post-traumatic stress disorder (PTSD) among emergency medical care workers in Riyadh, Saudi Arabia, as well as the primary causes of PTSD. The prevalence of PTSD in the case of emergency service practitioners was found to be 33.7% among all the research participants, which may be regarded as a high prevalence when compared to the general population. Additionally, gender, occupation, and working hours per week all demonstrated a significant difference. The study’s findings indicate that emergency medical personnel frequently suffer from PTSD. Given that our sample size was substantial, the results of this study might be applied to the situation facing other Saudi emergency medical service workers. In addition, our results may serve as evidence to assist practitioners, medical managers, experts, and technicians in determining the prevalence and incidence of PTSD among emergency care services units in Saudi Arabia. The knowledge about the prevalence of PTSD symptoms and ways to control the condition is crucial. The results of this research may benefit stakeholders in measuring the degree of psychological stress among emergency medical services practitioners encountered in the prehospital field. The assessment of the prevalence of PTSD may support establishing ways to diminish the level of stress among emergency medical services personnel in such a way as to enhance the quality of patient care, as well as improve the mental health of healthcare practitioners.

## Study limitations and suggestions for further research

The prevalence of PTSD can be impacted by both an increase in awareness of the condition and the occurrence of new traumatic events. Healthcare workers treating the influx of patients and working under high-stress conditions are at particular risk. Thus, it is important to periodically reassess the prevalence of PTSD among EMS workers. We measured the prevalence of PTSD at one medical facility in the city of Riyadh. Future studies should examine the incidence across a range of metropolitan centers, and extrapolate it to the national situation within Saudi Arabia, or to other nations that may participate in such a study. Secondly, we believe that a large sample size should be studied for various demographics including age, gender, and occupation. Equal sample sizes for men and women across all age categories should be used in future studies. Moreover, a comparison of various medical specialties and medical centers worldwide should be considered. We recommend that more research should be carried out regarding working experience, occupation, working hours, age and gender differences and prevalence of developing PTSD among emergency service personnel.

## Data availability statement

The original contributions presented in the study are included in the article/supplementary material. Further inquiries can be directed to the corresponding author.

## Ethics statement

The studies involving humans were approved by the Institute Review Board of King Saud University (IRB). The studies were conducted in accordance with the local legislation and institutional requirements. A consent form was attached to the questionnaire stating the purpose of the study and the contact information of the main author for any questions and/or inquiries. The identity of the participant was not obtained. Written informed consent was obtained from the individual(s) for the publication of any potentially identifiable images or data included in this article.

## Author contributions

SA: Writing – original draft, Software, Methodology, Investigation, Formal Analysis, Data curation, Conceptualization. AA: Writing – original draft, Supervision, Resources, Project administration, Investigation, Funding acquisition, Conceptualization. TA: Writing – review & editing, Writing – original draft, Visualization, Validation, Formal Analysis. JT: Writing – review & editing, Visualization. SR: Writing – review & editing, Visualization, Validation, Resources, Investigation, Formal Analysis.
